# Editorial: Embryo development and selection: advances in genetics

**DOI:** 10.3389/fmed.2024.1416791

**Published:** 2024-05-01

**Authors:** Zheng Liu, Yanlin Ma

**Affiliations:** ^1^College of Medical Laboratory Science, Guilin Medical University, Guilin, China; ^2^Hainan Provincial Key Laboratory for Human Reproductive Medicine and Genetic Research, Hainan Provincial Clinical Research Center for Thalassemia, Key Laboratory of Reproductive Health Diseases Research and Translation, Ministry of Education, Department of Reproductive Medicine, The First Affiliated Hospital of Hainan Medical University, Hainan Medical University, Haikou, Hainan, China

**Keywords:** assisted reproductive technologies, pregnancy rates, embryo development, embryo selection, influence factor

## Introduction

Assisted reproductive technologies (ART) are being utilized with increasing frequency across the globe to support individuals who face challenges in conceiving naturally. ART procedures facilitate pregnancy by extracting eggs from a woman's ovaries, fusing them with sperm in a laboratory setting, and subsequently reintroducing them into the woman's body. The efficacy of ART is subject to a multitude of factors, encompassing genetic considerations, historical medical and reproductive data, the specific medications employed during ART treatment, and any complications that may arise during pregnancy. The focus of this Research Topic is the selection of embryos during the ART process, and an exploration of the factors that influence embryo development and the overall success rate of ART.

## Key messages from the Research Topic

Abu et al. evaluated the impact of supplementing a single-dose GnRH agonist to the standard progestogen regimen for luteal phase support in IVF treatments. Compared to using progestogens alone, they found that adding a GnRH agonist improved the overall IVF outcomes.

Andreescu has conducted a review on the dysregulation of immune responses between the mother and fetus, a factor that heightens the risk of embryo rejection and reproductive failure. This articles provides an exhaustive overview of the current literature on the influence of Calcineurin inhibitors and anti-TNF treatment in enhancing the live birth rate post embryo transfer. It concludes that the suppression of immunological rejection and the promotion of immunological tolerance are vital in safeguarding embryos and averting immunological assaults. Therefore, it is important to exercise caution while selecting use of any immunosuppressive therapy in pregnancy.

Li et al. explore the potential of a new antioxidant, *N*-acetylcysteine, to enhance reproductive outcomes in older women undergoing *in vitro* fertilization/Intracytoplasmic sperm injection-embryo transfer (IVF/ICSI-ET). They examine its effect on the expression of L-Glutathione in follicular fluid and the mitochondrial DNA copy number in granulosa cells. Their research indicates that combining *N*-acetylcysteine with Gonadotropin-Releasing Hormone (GnRH) treatment may boost the ovarian response to superovulation drugs in ART, including in older demographics. Additionally, incorporating *N*-acetylcysteine during IVF procedures may enhance the quality of blastocysts in women of advanced age.

Yang et al. shed light on the results of expectant management for angular pregnancy after ART, laying the groundwork for the creation of clinical treatment strategies. Their study indicates that the risks associated with angular pregnancy following ART may not be as severe as previously thought. With regular, close monitoring, the majority of these cases can be managed expectantly, often resulting in live births.

Zhu et al. conducted a study to identify the risk factors that influence the occurrence of heterotopic pregnancies following IVF-ET. They also examined the outcomes of pregnancies after surgical intervention for heterotopic pregnancies. Their research indicates that a history of ectopic pregnancy, multiple abortions, tubal infertility, and multiple-embryo transfer may significantly increase the risk of a subsequent heterotopic pregnancy after IVF-ET. The study also found that for patients with heterotopic pregnancies who undergo surgery, factors such as a shorter operation duration, a smaller ectopic mass, and a location in the ampulla of the fallopian tube are associated with a more favorable reproductive prognosis.

## Building on current knowledge

IVF success rates are influenced by a multitude of factors, including genetic considerations, historical medical and reproductive data, the types of medications used during ART treatment, and complications that may arise during pregnancy. The research articles discussed here offer significant insights into improving the success rates of IVF. They underscore the efficacy of a combined treatment approach using *N*-Acetylcysteine and Gonadotropins, which has been shown to enhance ovarian response to superovulation drugs and improve the quality of blastocysts in older women. The articles also identify surgical time, the size of ectopic masses, and their location in the ampulla of the fallopian tube as key factors in determining the reproductive prognosis in patients with ectopic pregnancies. Additionally, the use of GnRH agonists in the luteal phase has been found to increase IVF success rates. The articles also discuss the evaluation of cornual pregnancy following ART. The aim of these studies is to reduce the risks associated with assisted reproduction and increase pregnancy rates, thereby providing invaluable information for clinical IVF centers.

## Conclusions

The referenced studies investigate the influence of several factors on the success rate of IVF. These factors encompass the patient's medical history, the treatment protocol, the medications administered during treatment, and complications encountered during pregnancy. The insights and research outcomes presented are of immense value for improving the success rates of assisted reproductive techniques.

## Author contributions

ZL: Writing – original draft, Writing – review & editing. YM: Conceptualization, Writing – review & editing.

